# Suppression of TCAB1 expression induced cellular senescence by lessening proteasomal degradation of p21 in cancer cells

**DOI:** 10.1186/s12935-020-01745-3

**Published:** 2021-01-07

**Authors:** Jing Niu, Rui-Qi Gao, Meng-Tian Cui, Chen-Guang Zhang, Shen-Tao Li, Shan Cheng, Wei Ding

**Affiliations:** 1 School of Basic Medical Sciences, Capital Medical University, 10 You’an Men West, Beijing, P. R. China; 2 Beijing Key Laboratory for Tumor Invasion and Metastasis Research, Capital Medical University, 10 You’an Men West, Beijing, P. R. China; 3 Central Facility of Biomedical Research, Capital Medical University, 10 You’an Men West, Beijing, P. R. China

**Keywords:** Cellular senescence, TCAB1, p21, Cancer, Proteasomal degradation

## Abstract

**Background:**

TCAB1, a.k.a. WRAP53β or WDR79, is an important molecule for the maintenance of Cajal bodies and critically involved in telomere elongation and DNA repair. Upregulation of TCAB1 were discovered in a variety types of cancers. However, the function of TCAB1 in tumor cell senescence remains absent.

**Methods:**

The TCAB1 knockdown cell lines were constructed. The expression levels of TCAB1, p21, p16 and p53 were detected by qRT-PCR and western blotting. Staining of senescence-associated β-galactosidase was used to detect senescent cells. The ubiquitination of the p21 was analysed by immunoprecipitation and in vivo ubiquitination assay. TCGA databases were employed to perform in silico analyses for the mRNA expression of TCAB1, p21, p16 and p53.

**Results:**

Here, we discovered that knockdown of TCAB1 induced rapid progression of cellular senescence in A549, H1299 and HeLa cells. In exploiting the mechanism underlining the role of TCAB1 on senescence, we found a significant increase of p21 at the protein levels upon TCAB1 depletion, whereas the p21 mRNA expression was not altered. We verified that TCAB1 knockdown was able to shunt p21 from proteasomal degradation by regulating the ubiquitination of p21. In rescue assays, it was demonstrated that decreasing the expression of p21 or increasing the expression of TCAB1 were able to attenuate the cellular senescence process induced by TCAB1 silencing.

**Conclusions:**

This study revealed the importance of TCAB1 for its biological functions in the regulation of cell senescence. Our results will be helpful to understand the mechanisms of senescence in cancer cells, which could provide clues for designing novel strategies for developing effective treatment regimens.

## Introduction

Cellular senescence is a state of irreversible growth arrest where cells remain viable and metabolically active, but non-proliferative even received mitogenic stimulations [1]. The senescence program can proceed in normal, preneoplastic, or malignant transformed conditions in cells exposed to various interruptions, such as telomere shortening, oncogene activation, oxidative stress, and DNA damages, etc. Although factors causing senescence may be different, the terminal senescence phenotype can be generally described as enlarged/flattened cell morphology, rise in activity of senescence-associated β-galactosidase (SA-β-gal), cell cycle arrest in G1 phase, and increased expression of cyclin-dependent kinase inhibitor (CDKI). At the molecular levels, senescence is governed by a chorus of multiple signaling pathways with dependence to distinct senescence stimuli. The induction and maintenance of senescence predominantly involves two major pathways, which are denoted by critical tumor suppressors as the p16^INK4a^/pRb and p53/p21 pathways [2]. It is believed that the establishment of cell senescence will drive cancer cells to (re-)entry irreversible cell cycle arrest [3]. Therefore, the induction of tumor cell senescence is perceived as a promising anti-cancer strategy for developing novel therapies [4].

The regulation of cellular senescence often requires the function of p21 as a key mediator molecule for signal transduction. By blocking the kinase activity of CDKs, the CDK2 inhibitor p21 is an important cellular factor to brake G1 progression and induce senescence. The expression of p21 is regulated by complex mechanisms during the process of cellular senescence, including epigenetic silencing, transcriptional or translational controls, and protein stability changes from ubiquitin dependent or independent degradation [5]. As a major regulatory transcriptional factor of p21, p53 mutation is associated with decreased p21 expression. The p53-independent induction of p21 has also been reported. Ubiquitin-dependent degradation of p21 requires several E3 ligases, such as SCF^skp2^, CRL4^cdt2^, and APC/C^cdc20^ [6–8], which functions at different stages of the cell cycle. Other E3 ligases of FBXO22 and CHIP was also found to involve p21 degradation through ubiquitin-mediated mechanisms [9, 10]. Interactions with certain proteins can stabilize p21 and prevent its ubiquitin-independent degradation [11].

TCAB1 is a structural and essential component protein of the Cajal body (CB). Through its WD40 domains and the c-terminal region containing 456 to 533 amino acids, TCAB1 interacted with Coilin and SMN proteins [12]. TCAB1 is crucial in the synthesis of telomeres in human tumor cells, and is able to transport telomerase directly to telomeres to facilitate terminal elongation [13]. TCAB1 overexpression can help to clear γH2AX from ionizing radiation and promote the efficiency of homologous recombination and non-homologous end-joining repairs [14]. Dysfunction of TCAB1 has been linked to congenital dyskeratosis, spinal muscular atrophy, tumors, premature aging. Abnormal TCAB1 expression has been observed in nasopharyngeal [15], esophageal[16], rectal [17], breast [18], ovarian cancers [19], and recently in non-small cell lung cancer (NSCLC) as well[20]. The role of TCAB1 in tumors may related to impaired telomere function and impaired recruitment of shear factors in pre-mRNAs. TCAB1 overexpression promotes cell proliferation, whereas silencing the TCAB1 gene significantly inhibited the growth by inducing cell cycle arrest and apoptosis.

In this study, we find that knocking down TCAB1 in stable cancer cells induced striking cellular senescence phenotype. The effect of TCAB1 silencing was mediated by decreased ubiquitination of p21 and its subsequent proteasome-mediated degradation. Removal of cumulated p21 under TCAB1 deficiency allowed the cells to escape from senescence. Taken together, we have identified a new biological function of TCAB1 for its involvement in cellular senescence. Our findings suggested that reducing TCAB1 could be deployed as a valid strategy to develop anticancer treatment as it appeared to be sufficiently to trigger cellular senescence.

## Materials and methods

### Tissue culture and establishment of stable cell lines

A549, H1299 and HeLa cells were obtained from the cell bank at Peking Union Medical University and were detected for STR profiles in china. Cells were grown in Dulbecco’s modified Eagle’s medium (DMEM, HyClone) supplemented with 10% fetal bovine serum (HyClone) and 100 units/ml penicillin and streptomycin in a humidified incubator with 5% CO_2_ at 37 °C. The RNA interference target sequences corresponding to TCAB1 (shRNA: TATCTGGGACGCATTCACT) and a scrable sequence (TTCTCCGAACGTGTCACGT) were subcloned into lentiviral vectors of GV152 backbone (vector containing a puromycin resistance gene; Genechem). The lentiviral of LV-TCAB1 were provided by Hesheng genomic technology. The cells were infected with the produced lentivirus in the presence of polybrene (8 μg/ml; Sigma) for 48 h, and then selected by 2 μg/ml puromycin (Sigma) for 7 days. The expression levels of the targeted gene were determined by RT-PCR and western blotting for knockdown efficiencies.

### RNA extraction and qRT-PCR

Total RNA was isolated from HeLa stable cells using the RNeasy mini kit (Qiagen) following to the manufacturer’s standard protocol. The RNA concentrations and purity were determined using a NanoDrop 2000 spectrophotometer (Thermo Fisher Scientific). A total of 1 μg of RNA each was subjected to reactions to obtain cDNAs using a Reverse Transcription Kit (Promega). Quantitative Real-Time-PCR was performed by an ABI 7500 Real-Time PCR System (Applied Biosystems) using Maxima SYBR Green/ROX qPCR Master Mix (MBI Fermentas). Thermal cycling conditions were 95 °C for 30 s, followed by 5 s at 95 °C and 1 min at 60 °C for 40 cycles. PCR amplification was performed using specific primers: p21 (CGAGACACCACTGGAGGGT; R: GAGGCACAAGGGTACAAGACA); TCAB1 F: (F: TCAAGAAGGTGGTGAAGCA; R: GTCAAAGGTGGAGGAGTGG); and GAPDH (F: CAAGGCTGTGGGCAAGGT; R: CCTGCTTCACCACCTTCTT). Relative quantities of mRNA were calculated using the comparative Ct method for each sample. Analyses were done in triplicate to confirm the data.

### Western blotting

The cell culture samples were lysed in modified Radioimmune Precipitation Assay (RIPA) Buffer (Applygen) containing a protease inhibitor cocktail (Roche). The protein concentrations were measured using the BCA assay kit (Thermo Fisher Scientific). A total protein of 10–20 μg from each sample were loaded and subjected to 10% sodium dodecyl sulfate polyacrylamide gel electrophoresis (SDS-PAGE), and then transferred to PVDF filters (Millipore). Immunoprobing of target proteins were performed by incubating overnight at 4 °C with the primary antibodies against p21 (#10355–1-AP, Proteintech), TCAB1(#14761–1-AP, Proteintech) or ubiquitin (#D058-3, MBL). After rinses, HRP-conjugated secondary antibodies used for western blotting analysis were as follows: anti-rabbit IgG (#3012; Signalway Antibody) and mouse IgG (#3032; Signalway Antibody). The even loading of isolated proteins was verified with β-actin monoclonal antibody (#3700S, Cell Signaling Technology) or GAPDH monoclonal antibody (#10494–1-AP, Proteintech). The blotted filters were visualized for signals using enhanced chemiluminescence (ECL) reagents (GE Healthcare) according to the manufacturer’s instructions.

### Staining of senescence-associated β-galactosidase

For SA-β-gal staining, cells were seeded into 12-well plates and cultured in a DMEM medium. After fixed for 20 min at room temperature in 4% paraformaldehyde, the cells were washed twice with 1× PBS. A freshly prepared SA-β-gal staining solution was used to incubated with the fixed cells overnight at 37 °C with the absence of CO_2_. Eight randomly selected representative fields were quantified for the percentage of SA-β-gal positive cells. Data were presented as mean ± SE.

### *Immunoprecipitatation and *in vivo* ubiquitination assay*

To detect the levels p21 ubiquitination, A549-shTCAB1, H1299-shTCAB1 and shTCAB1 stable HeLa cells were pretreated with 20 μM MG132 (Merck Millipore) for 6 h. The whole cell lysates were collected from 10 cm dishes and incubated with anti-p21 antibodies overnight at 4 °C. Then, 20 μl beads conjugated with protein A/G were added and incubated for 1 h at room temperature. The beads were washed three times with the washing buffer (10 mM Tris·HCl, pH 8.0, 1 M NaCl, 1 mM EDTA, 1% Nonidet P-40) prior to immunoprecipitation. The released samples in 20 μl 2× SDS protein loading buffer were subjected to western blotting analyses using an antibody against ubiquitin.

### Protein half-life determination

The transformed stable cells were treated with 100 μg/ml of cycloheximide for 0, 0.5, 1, 2, 3, 4 and 5 h. The whole cell lysates were prepared. The amount of 10 or 20 μg of total protein from each sample was analyzed by Western blotting with an anti-p21 antibody.

### RNAi and transfection

To transiently silence p21, siRNA targeting p21 was synthesized (Hesheng genomic technology) and transfected with Lipofectamine™ RNAiMAX (Invitrogen) following the manufacturer’s instructions. The siRNA sequence target p21 was: CGUCAGAACCCAUGCGGCATT. Control insertion sequence was: UUCUCCGAACGUGUCACGUT.

### 3D terrain map

From TCGA database, the mRNA expression of TCAB1, p21 and p16 of a total of 514 lung cancer patients were retrieved. For better and fair comparison of genes of different expression levels and abundance, the expression levels of each gene were normalize by quartile ranking scales and classified into 9 group from low to high as “0 to 8”. Using the normalized levels as the x and y scales, TCAB1/p21 or TCAB1/p16 in our case, the averaged level of a third gene of interest (p53 in this study) could be ploted with the original raw values to generate a 3D terrain map. Both p53 wild-type (362) and p53 mutation (152) cases were analyzed and presented as comparative panels.

## Results

### RNA interference of TCAB1 induced cellular senescence

**Fig.1:**
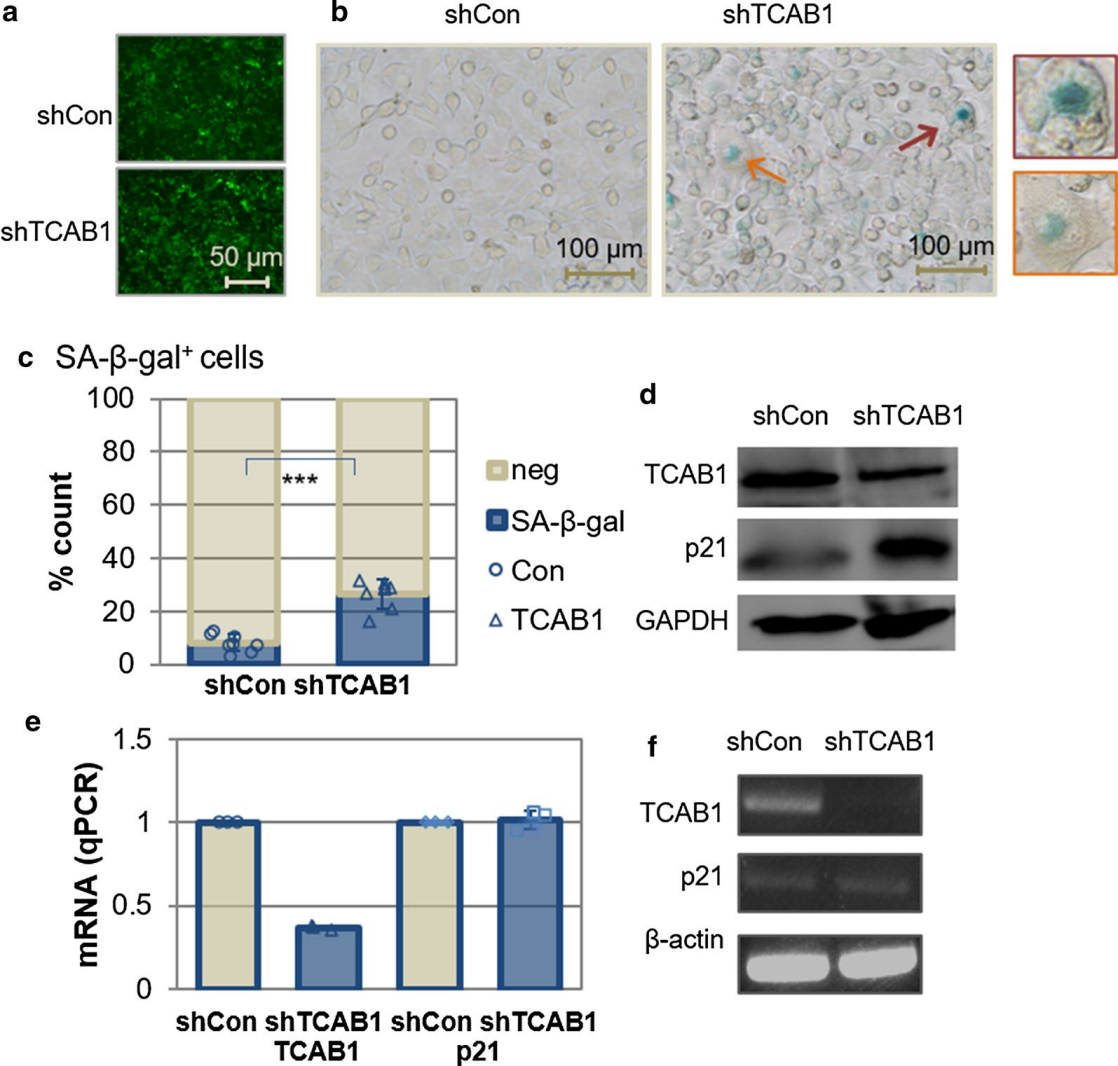
RNA interference of TCAB1 induced cellular senescence in HeLa cells. **a** Lentivirus of shTCAB1 or shCon transformed HeLa cells were prepared by lentiviral infections. The efficiency of lentivirus infection was observed by fluorescence microscope. **b** Cellular senescence was assayed by senescence-associated (SA)-β-gal staining (arrows pointing to cells with representative senescent phenotype). **c** Quantification from B for SA-β-gal positive cells in percentages (n = 8). **d** The protein levels of TCAB1 and p21 were determined by Western blotting and compared to those in the control cells. **e** The mRNA expression levels of TCAB1 and p21 were measured by qRT-PCR analysis (n = 3). **f** The RT-PCR products of TCAB1 and p21 detected by agarose electrophoresis

TCAB1 is a scaffold protein that directs factors to Cajal bodies and participates in DNA damage repair. Cajal body is extremely sensitive to DNA damage and is associated with chemotherapy-induced tumor cell senescence [21]. TCAB1 was shown to be overexpressed in both cancer cells and neoplastic tissues, whereas downregulation of TCAB1 inhibited tumor cell proliferation. These let us sought to explore on whether TCAB1 has a significant impact on the process of cellular senescence. HeLa cells is a representative model for studying tumor cell senescence due to its better characterized genetic background and easy observation for senescence phenotype development. We infected HeLa cells with lentiviruses to silence TCAB1 expression. After 1 week selection with puromycin, almost all the cells were infected with shTCAB1 (Fig. 1a) and the cells were analyzed for SA-β-gal activity. We found that a significant portion of selected cells developed senescence-like phenotype as manifested by enlarged and flattened morphology (Fig. 1b). The SA-β-gal^+^ cells were observed significantly increased in numbers as compared to cells infected with the control virus (Fig. 1c). As p21 is a known important gene to drive the process of cellular senescence, we wonder if the p21 expression was altered in shTCAB1 transformed cells. Indeed, the level of p21 proteins significantly upregulated in HeLa cells with the silencing of TCAB1 (Fig. 1d). We next to examine if the increase in p21 expression following TCAB1 knockdown occured at the transcription or posttranslation levels. To our surprise, there were no significant changes of p21 mRNA in these cells when TCAB1 were knockdown as shown in Fig. 1e, f.

### Silencing of TCAB1 stabilized p21 from proteasomal degradation

**Fig.2:**
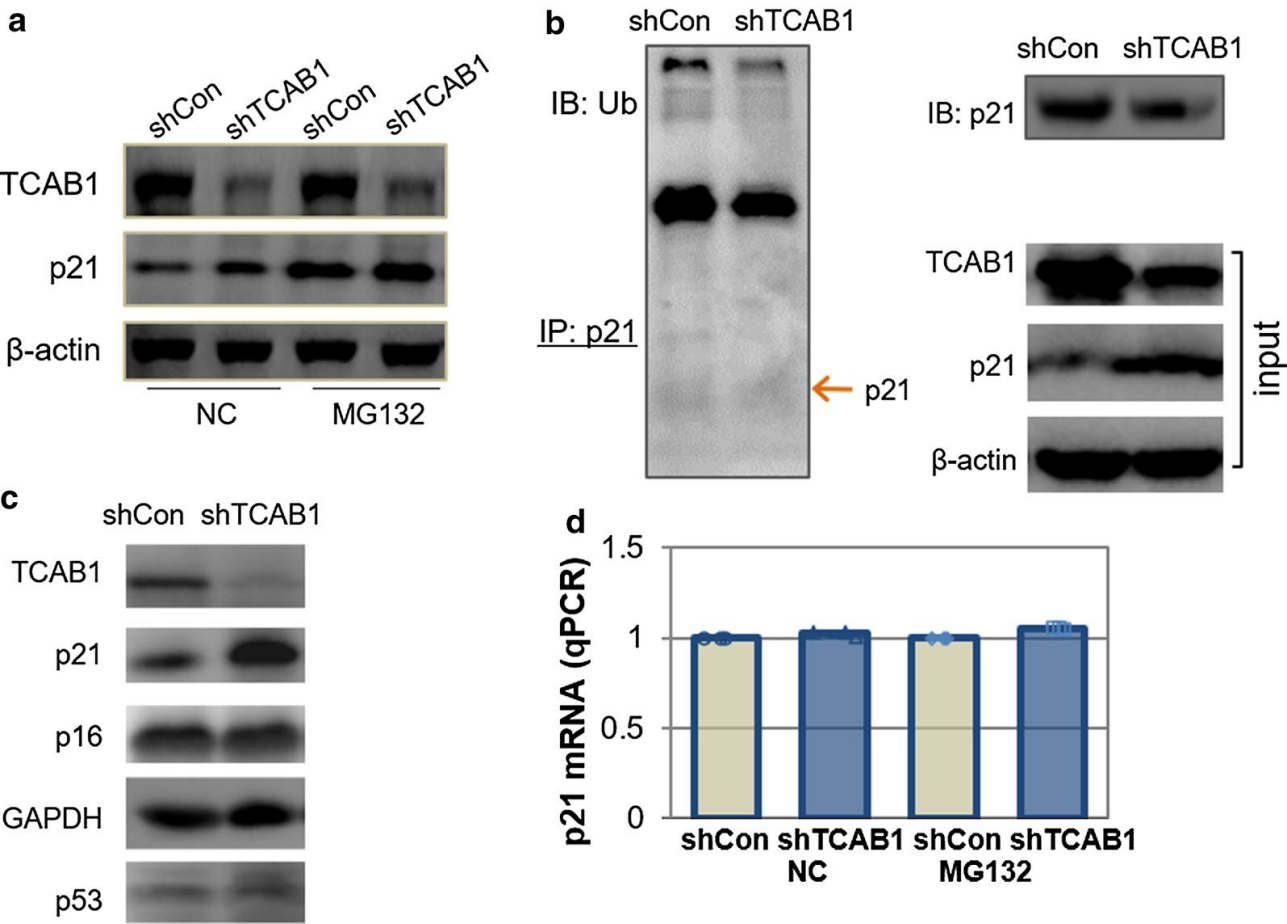
Knockdown of TCAB1 inhibited the degradation of p21 in HeLa cells. **a** HeLa cells infected with shTCAB1 lentivirus following DMSO (NC) or MG132 (20 μM) treatments for 6 h. The protein levels of TCAB1 and p21 were detected by western blotting. **b** The shTCAB1 transformed HeLa cells from lentiviral infections were treated with MG132 (20 μM) for 6 h. Cell Lysates were immunoprecipitated with anti-p21 antibody and analyzed by western blotting using an anti-ubiquitin antibody. **c** The protein levels of TCAB1, p53, p21and p16 were determined by Western blotting in shTCAB1 lentivirus infected cells and the control cells. **d** The mRNA expression levels of p21 were measured by qRT-PCR in HeLa infected with shTCAB1 lentivirus after treated with DMSO (NC) or MG132 (20 μM)

Degradation of p21 through the ubiquitin proteasome system is one of the major ways to regulate p21 protein levels. The HeLa cells steadily knockdown TCAB1 were treated with the proteasome inhibitor MG132. As shown in Fig. 2a, in the absence of MG132, p21 protein levels increased obviously accompanied by TCAB1 downregulation in HeLa cells. Such effect of TCAB1 on p21 could be reversed by the proteasome inhibitor MG132, suggesting that TCAB1 regulated p21 levels in a proteasome-dependent manner. To further verify degradation of p21 involved the regulation of p21 ubiquitination, cells were treated with the proteasome inhibitor MG132 were detected for polyubiquitinated p21 proteins and their accumulation. As shown in Fig. 2b, p21 ubiquitination was significantly decreased when TCAB1was knocked down in HeLa cells. It has been known that p16 is also a key inducer of cell senescence. In addition to p21, we analyzed of p16 protein levels in Fig. 2c and found no significant changes when knockdown of TCAB1. To exclude the possibility of MG132 treatment might directly alter the mRNA level of p21, we measured the mRNA level of p21 in MG132 treated cells. We found that the p21 mRNAs appeared to be stable in cells with or without exposure to MG132 (Fig. 2d). Taken together, these findings suggest that TCAB1 controls the stability of p21 by modulating their ubiquitination prior to proteasomal degradation.

### Knockdown of TCAB1 expression induced senescence by regulating the ubiquitination of p21 in A549 and H1299 cancer cells

**Fig.3:**
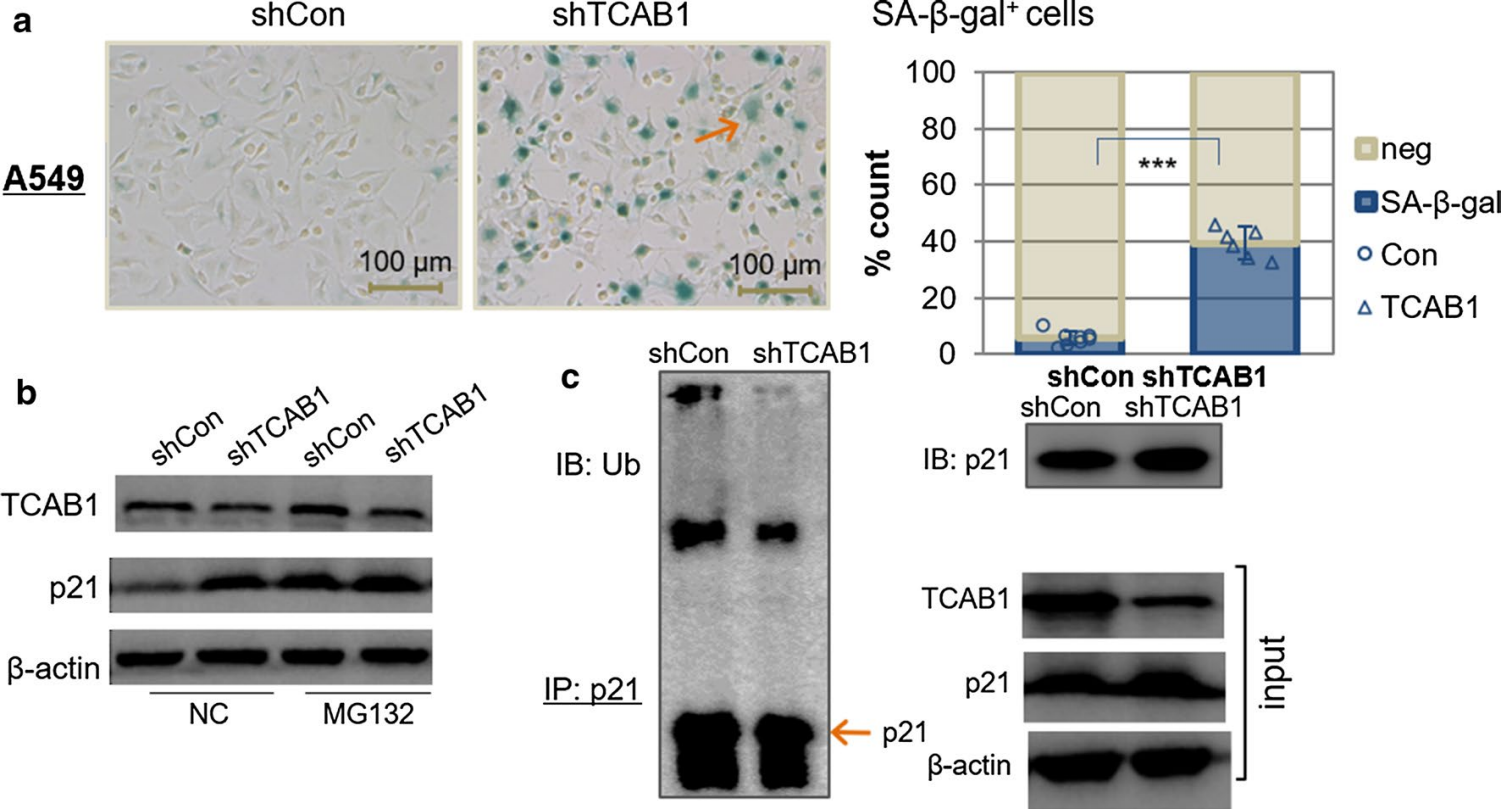
Suppression of TCAB1 expression induced senescence by regulating the ubiquitination of p21. **a** A549 cells were infected with shTCAB1 or shCon lentivirus. Cellular senescence was measured by Senescence-associated (SA)-β-gal assay (arrows pointing to cells with representative senescent phenotype). The percentage of cells positive for (SA)-β-gal was quantized (n = 8). **b** A549 cells infected with lentivirus encoding the indicated shRNA were treated with DMSO or MG132 (20 μM) for 6 h. The protein levels of TCAB1 and p21 were detected by western blotting. **c** The shTCAB1 transformed A549 cells from lentiviral infections were treated with MG132 (20 μM) for 6 h. Lysates were immunoprecipitated with anti-p21 antibody. The ubiquitination of the p21 was analysed by western blotting using antibody against ubiquitin. **d**–**f** Senescence phenotypes and the degradation and p21 ubiquitination were detected in TCAB1 knockdown H1299 cells

Because TCAB1 is overexpressed in human non-small cell lung cancer tissues and cell lines, we next to explore whether depletion of TCAB1 could induce cellular senescence in the lung cancer cells. The major difference between A549 and H1299 cells is the existence of wild type p53 molecules. We suspected the drastic difference in senescent phenotype of these two cell lines might involve the participation of p53 proteins. The state of p53 is known to influence cellular senescence process, and mutations in p53 are most frequently observed in cancer cells. Many tumors have inactive p53, which results in resistance to both apoptosis and senescence [22].We had observed previously that silencing of TCAB1 induced senescence in HeLa cells which has low p53 expression*.* Therefore, we wanted to know if downregulation of TCAB1 in wild type p53 and p53-deficient cells also sensitized them to senescence. When we silenced TCAB1 gene in A549 (wild type p53) cells, we found that the absence of TCAB1 could induce senescence dramatically. Nearly half of the cells were stained positive by SA-β-gal (Fig. 3a). Similarly, we wanted to investigate whether inhibition of p21 degradation caused by downregulation of TCAB1 was related to p53. As shown in Fig. 3b, c, suppression of the expression of TCAB1 also protected the degradation of p21 from the proteasome pathway and reduced the ubiquitination level of p21 in A549 cells. When we downregulated TCAB1 gene in H1299 (p53−/−) cells, we also found that the depletion of TCAB1 could induce senescence and repress the degradation of p21 from the proteasome pathway (Additional file 1: Fig. S1A–C). Although inhibition of TCAB1 induced a senescent phenotype in all three cell lines, senescent cells were induced most frequently in A549 cells. In summary, it can be concluded that TCAB1 knockdown induces tumor cell senescence by regulating the ubiquitination and degradation of p21. In wild-type p53 tumor cells, the senescence phenotype induced by the deletion of TCAB1 is more obvious.

### Depletion of p21 attenuated senescence induced by TCAB1 knockdown

**Fig.4:**
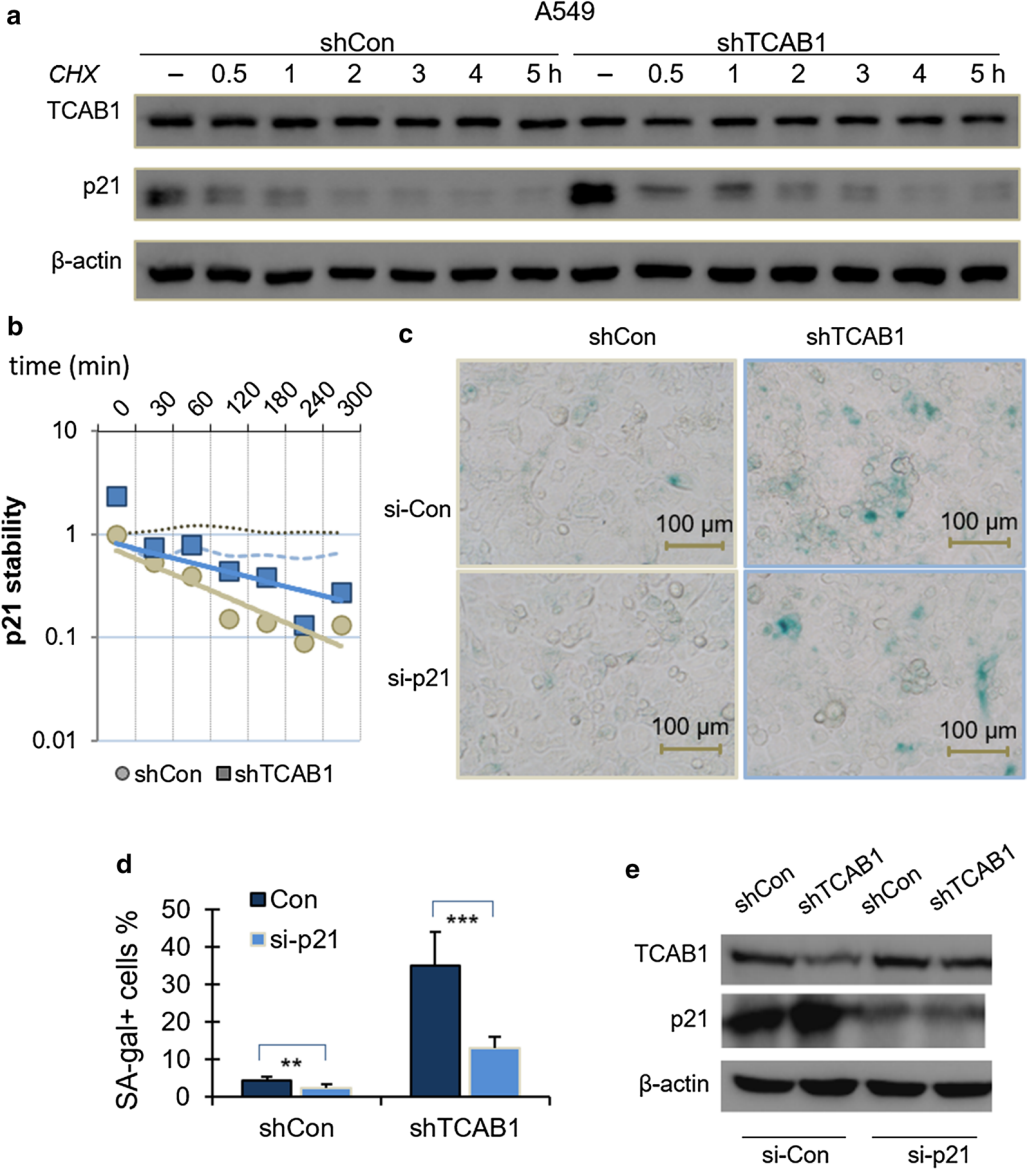
Depletion of p21 in TCAB1 knockdown cells attenuated the development of senescence phenotype. **a** To determine the half-life of p21, A549 cells were treated with cycloheximide (100 μg/ml) for indicated periods of time, and then immunoblotted with probing antibodies. **b** Quantification of the p21 protein levels relative to β-actin. Intensities of blots were analysed by the ImageJ software. **c** SA-β-gal staining in shTCAB stable A549 lung cancer cells transfected with either a scramble control or p21 siRNA. **d** Quantification from C for SA-β-gal positive cells in percentages (n = 8). **e** The protein levels of TCAB1 and p21 determined by western blotting

Since p21 is a protein with relative short half-life, we predicted that the effect of TCAB1 on p21 degradation could be monitored in kinetic assays of protein stability. Because the senescence phenotype induced by the downregulation of TCAB1 is more obvious in A549 cells, we next investigated whether TCAB1 could regulate p21 stability in A549 cells. A549 cells with or without TCAB1 knockdown were treated with cycloheximide (CHX) to inhibit protein biosynthesis, and protein extracts obtained at the indicated time points were analyzed. We found that knockdown of TCAB1 in cells resulted in a significantly extended half-life of p21 (Fig. 4a, b). To further determine whether TCAB1 knockdown induced senescence through the ubiquitination of p21, we ectopically knocked down p21 in TCAB1-delepted A549 cells and verified the protein depletion by western blot analysis. Importantly, the ectopic knockdown of p21, but not the control, significantly reversed senescence of A549 cells with low expression of TCAB1 (Fig. 4c, d). Western blotting results showed that TCAB1 and p21 were knocked down in shTCAB1 infected and sip21 treated cells (Fig. 4e). Taken together, these results indicated that inhibition of p21 could rescue knockdown of TCAB1 induced senescence.

### Overexpression of TCAB1 reversed senescence induced by TCAB1 knockdown

**Fig.5:**
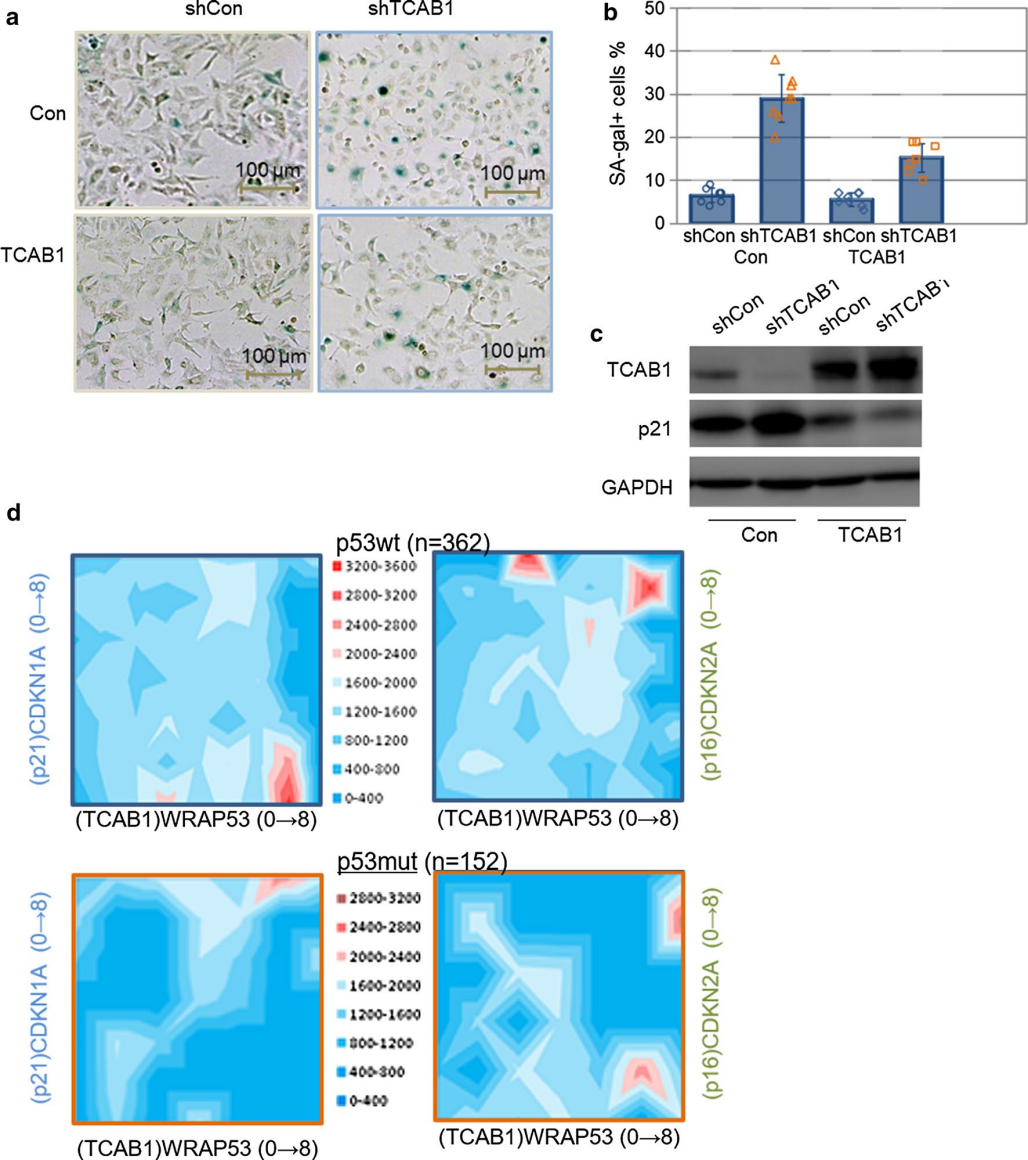
Overexpression of TCAB1 prevented senescence by regulating the expression of p21. **a** A549 cells stable expressed shRNA against TCAB1 were infected with LV-TCAB1 lentivirus and control lentivirus. Cellular senescence was measured by Senescence-associated (SA)-β-gal assay. **b** Quantification from A for SA-β-gal positive cells in percentages (n = 8). **c** Western blotting were used to determine TCAB1 and p21 expression. GAPDH was loading control. **d** RNAseq results of TCAB1, p21, p16 and p53 levels in lung adenocarcinoma patients of both p53 wild type (362 cases) and mutant p53 (152 cases) groups from cBioPortal (Luad_tcga_pan_can_atlas_2018) were summarized in 3D terrain charts

In order to further explore the effect of TCAB1 on senescence, we overexpressed LV-TCAB1 or control lentivirus in TCAB1-delepted A549 cells. We found that overexpression of TCAB1 can partly reversed the senescence phenotype induced by depletion of TCAB1. The positive rates of cells staining SA-β-gal was significantly lower in TCAB1 overexpressing cells than the control (Fig. 5a, b). Western blotting results showed that TCAB1 were overexpressed and p21 were repressed in LV-TCAB1treated cells (Fig. 5c). To further explore the clinical significance of TCAB1 and senescence related important proteins, we analyzed the mRNA expression of TCAB1, p21, p16 and p53 in 514 patients with lung cancer including p53 wild-type (362) and p53 mutation (152) in the TCGA database. In patients with wild-type p53 as shown in the upper-left pane in Fig. 5d, more samples (reflected by the grid density) were with high TCAB1 expression (to the right side of the scale), which demonstrated that in tumors TCAB1 were often upregulated. However, we noticed in highest TCAB1 samples, there were 2 distinguishable groups, where low p21 expression ones showing the highest p53 expression. Such a pattern were not seen when profiling with p16 (or perhaps even reversed) shown in upper-right pane in Fig. 5d. In addition, a clear difference can be observed from same analyses in p53 mutant sample (Fig. 5d lower panes). Our previous results (Fig. 3a) also suggested that in wild-type A549 cells, the senescence phenotype induced by knocking down TCAB1 was most pronounced. These results suggest that TCAB1 intervention may have a good role in inducing cell senescence and inhibiting tumor in wild-type p53 lung cancer.

## Discussion

TCAB1 was demonstrated as an important structural component of Cajal body, facilitates interactions of factors involved in splicing and telomere elongation and their assembly in Cajal bodies [12]. TCAB1 is frequently overexpressed in a variety of tumor cells, and its loss or abnormally high expression can cause CB disintegration and mislocalization of related factors. We previously reported that blocking the formation of CB by knocking down coilin promoted the senescence phenotype, demonstrated in HeLa cells treated with cisplatin [21]. We also found in leukemic cells that knocking down coilin decreased the expression of the p27 protein [23].Therefore, we hypothesized the expression of TCAB1 could be a critical determinant of cell senescence. In this study, we provided evidence that knockdown of TCAB1 in cancer cells bearing either wild-type or deletion of p53 significantly increased p21 protein and subsequently induced cellular senescence in a subset of cells. It is possible that this phenomenon is related to the DNA repair capacity in tumor cells. Several studies suggested that DNA damage is an important regulator of cell senescence, which triggers a DNA damage response (DDR). Pathways that allow cells to sense damaged DNA and respond by repairing damage or by making arrests cell cycle progression [24]. Persistent DDR signaling is critical initiation and maintenance of aging [25]. The depletion of TCAB1 may induce DNA damage which is a major cause of cellular senescence. It was reported that TCAB1 RNAi in U2OS, H1299 and HeLa cells increased the number of phospho-histone H2AX (γ-H2AX) foci in nonirradiated cells, indicating TCAB1 protects cells against accumulation of spontaneous DNA damage [14]. Cells overexpressing TCAB1 exhibited more rapid clearance of γ-H2AX, in association with homologous recombination and non-homologous end-joining at sites of less DNA breaks [26]. Reduced expression of TCAB1 in ovarian tumors correlated with attenuated DNA damage response and poor patient survival [19]. Based on these findings, we ration that one major driving force behind shTCAB-induced senescence in tumor cells may relate to insufficiency of DDR and the impaired DNA repair ability, which tends to result more severe DNA damages and genomic instability and cause irreversible senescence.

In mammalian cells, the senescence checkpoint is initiated by one of the two major signaling pathways p21 /p53 and p16/Rb. We demonstrated that TCAB1 reduction-induced senescence was not dependent to p16 expression, rather signatured by an increase at p21 protein levels. Elevated levels of p21 in TCAB1-depleted cells resulted from avoiding the degradation from ubiquitin proteasome system. The mechanism of p21 degradation is currently not fully understood. It is believed that the degradation of p21 could be through either ubiquitin-dependent or independent mechanisms. Newly synthetized p21 is stabilized by WIS39, an adaptor that recruits HSP90 to p21 [27] while p21 ubiquitylation and degradation are promoted by several E3 ubiquitin ligase, such as SCF^SKP2^ ubiquitin ligase, the CRL4^CDT2^ ligase, and the APC/C^CDC20^ ubiquitin ligase. Even though p21 is polyubiquitylated in cells, ubiquitylation is not always required for p21 degradation, because a p21 mutant with all lysine residues mutated to arginine (p21K0) can still be degraded [28, 29]. At present study, the data suggested that the scaffold protein TCAB1 interfere with the ubiquitination of p21 and sufficiently alter the cellular senescent phenotype. This finding added TCAB1 to the growing list of protein regulating p21 protein abundance, and emphasized the potentially role of Cajal bodies for their involvement in the regulation of senescence. Bearing six individual WD-repeat domains, TCAB1/WDR79 belongs to the WD-repeat protein family. The WDR domains are often essential subunits of multi protein complexes involved in a wide range of cellular processes, including the protein ubiquitination, gene expression, signal transduction, histone methylation and cell cycle control [30]. We hypothesized that TCAB1 regulated ubiquitination degradation of p21 through the interaction between its WDR domain and ubiquitin ligase.

Another important finding from this study was that A549 lung cancer cells appeared to be much more sensitive to TCAB1 suppression for senescence induction. As A549 contains the wild type of p53, it could explain, at least partially, our discovery indicating the p21/p53 pathway was responsible for TCAB1 participated senescence regulation. Surprisingly, in the wild-type p53 patients analyzed in the TCGA database, the expression of TCAB1 and p21 in patients with high p53 expression was negatively correlated. This turned out to be reasonable to explain that why p21/p53 pathway for cell senescence do not activate in tumor cells, even when p53 functions are intact. The interconnections among the state and expression of p53 and TCAB1/p21 could be of great biological significance and remains to be further studied. It should be pointed out that the TCGA results are gene expression measures at the mRNA levels, even in support of our hypothesis on TCAB1 and p21 axis on senescence regulation, more direct evidence from assays on TCAB1 and p21 protein levels from clinical samples will add more convincing values.

## Conclusions

In summary, we observed that knockdown of TCAB1 in cancer cells sufficiently induced cellular senescence of different extent, possibly depending on the status of p53 gene. TCAB1 knockdown was able to increase the p21 protein level by reducing its proteasomal degradation through ubiquitination dependent fashion. In A549 cells with wild type p53, the phenotype of TCAB1-KD induced senescence was more pronounced with p21 dependency as demonstrated by si-p21 rescue experiments. However, whether TCAB1 deletion can induce tumor senescence in vivo remains to be explored. Molecule TCAB1 interacts directly with to influence the ubiquitination of p21 needs further study.

Cellular senescence is a prominent tumor suppression strategy to allow cancer cells escape from malignant expansion and development of treatment resistance. Induction of senescence in cancer cells is emerging as a practical therapeutic concept [31]. Depletion of TCAB1 is crucial for ensuring the irreversibility of the senescence arrest in p53 wild type and mutant cells. Given the fact that more than half of the tumors maintained the p53 wild type forms, including lung cancers with the most incidences, our findings not only offer new perspectives in the modulation of senescence by TCAB1 but also suggest a novel therapeutic target for cancer treatment, especially for patients that might failing chemotherapies with risks of over doses.

## Supplementary Information

**Additional file 1. Fig.S1 A-C**

## Data Availability

All data generated or analysed during this study are included in this published article.

## References

[1] Campisi J, di Fagagna FD (2007). Cellular senescence: when bad things happen to good cells. Nat Rev Mol Cell Bio.

[2] Campisi J (2013). Aging, cellular senescence, and cancer. Annu Rev Physiol.

[3] Collado M, Blasco MA, Serrano M (2007). Cellular senescence in cancer and aging. Cell.

[4] Leonart ME, Arteroastro A, Kondoh H (2009). Senescence induction; a possible cancer therapy. Mol Cancer..

[5] Jung YS, Qian Y, Chen X (2010). Examination of the expanding pathways for the regulation of p21 expression and activity. Cell Signal.

[6] Bornstein G, Bloom J, Sitry-Shevah D, Nakayama K, Pagano M, Hershko A (2003). Role of the SCFSkp2 ubiquitin ligase in the degradation of p21Cip1 in S phase. J Biol Chem.

[7] Abbas T, Sivaprasad U, Terai K, Amador V, Pagano M, Dutta A (2008). PCNA-dependent regulation of p21 ubiquitylation and degradation via the CRL4Cdt2 ubiquitin ligase complex. Genes Dev.

[8] Amador V, Ge S, Santamaria PG, Guardavaccaro D, Pagano M (2007). APC/C(Cdc20) controls the ubiquitin-mediated degradation of p21 in prometaphase. Mol Cell.

[9] Biswas K, Sarkar S, Du K, Brautigan DL, Abbas T, Larner JM (2017). The E3 Ligase CHIP mediates p21 degradation to maintain radioresistance. MCR.

[10] Zhang L, Chen J, Ning D, Liu Q, Wang C, Zhang Z, Chu L, Yu C, Liang HF, Zhang B (2019). FBXO22 promotes the development of hepatocellular carcinoma by regulating the ubiquitination and degradation of p21. J Exp Clin Cancer Res.

[11] Xu S, Feng Z, Zhang M, Wu Y, Sang Y, Xu H, Lv X, Hu K, Cao J, Zhang R (2011). hSSB1 binds and protects p21 from ubiquitin-mediated degradation and positively correlates with p21 in human hepatocellular carcinomas. Oncogene.

[12] Mahmoudi S, Henriksson S, Weibrecht I, Smith S, Soderberg O, Stromblad S, Wiman KG, Farnebo M (2010). WRAP53 is essential for Cajal body formation and for targeting the survival of motor neuron complex to Cajal bodies. PLoS Biol.

[13] Venteicher AS, Abreu EB, Meng Z, McCann KE, Terns RM, Veenstra TD, Terns MP, Artandi SE (2009). A human telomerase holoenzyme protein required for Cajal body localization and telomere synthesis. Science.

[14] Henriksson S, Rassoolzadeh H, Hedstrom E, Coucoravas C, Julner A, Goldstein M, Imreh G, Zhivotovsky B, Kastan MB, Helleday T (2014). The scaffold protein WRAP53beta orchestrates the ubiquitin response critical for DNA double-strand break repair. Genes Dev.

[15] Sun CK, Luo XB, Gou YP, Hu L, Wang K, Li C, Xiang ZT, Zhang P, Kong XL, Zhang CL (2014). TCAB1: a potential target for diagnosis and therapy of head and neck carcinomas. Mol Cancer.

[16] Rao X, Huang D, Sui X, Liu G, Song X, Xie J, Huang D (2014). Overexpression of WRAP53 is associated with development and progression of esophageal squamous cell carcinoma. PLoS ONE.

[17] Zhang H, Wang DW, Adell G, Sun XF (2012). WRAP53 is an independent prognostic factor in rectal cancer—a study of Swedish clinical trial of preoperative radiotherapy in rectal cancer patients. BMC Cancer.

[18] Silwal-Pandit L, Russnes H, Borgen E, Skarpeteig V, Moen Vollan HK, Schlichting E, Karesen R, Naume B, Borresen-Dale AL, Farnebo M (2015). The sub-cellular localization of WRAP53 has prognostic impact in breast cancer. PLoS ONE.

[19] Hedstrom E, Pederiva C, Farnebo J, Nodin B, Jirstrom K, Brennan DJ, Farnebo M (2015). Downregulation of the cancer susceptibility protein WRAP53beta in epithelial ovarian cancer leads to defective DNA repair and poor clinical outcome. Cell Death Dis.

[20] Sun Y, Yang C, Chen J, Song X, Li Z, Duan M, Li J, Hu X, Wu K, Yan G (2016). Overexpression of WDR79 in non-small cell lung cancer is linked to tumour progression. J Cell Mol Med.

[21] Song Y, Niu J, Yue Z, Gao R, Zhang C, Ding W (2017). Increased chemo-sensitivity by knockdown coilin expression involved acceleration of premature cellular senescence in HeLa cells. Biochem Biophys Res Commun.

[22] Ventura A, Kirsch DG, McLaughlin ME, Tuveson DA, Grimm J, Lintault L, Newman J, Reczek EE, Weissleder R, Jacks T (2007). Restoration of p53 function leads to tumour regression in vivo. Nature.

[23] Yue ZX, Gao RQ, Gao C, Liu SG, Zhao XX, Xing TY, Niu J, Li ZG, Zheng HY, Ding W (2018). The prognostic potential of coilin in association with p27 expression in pediatric acute lymphoblastic leukemia for disease relapse. Cancer Cell Int.

[24] Bartkova J, Rezaei N, Liontos M, Karakaidos P, Kletsas D, Issaeva N, Vassiliou LV, Kolettas E, Niforou K, Zoumpourlis VC (2006). Oncogene-induced senescence is part of the tumorigenesis barrier imposed by DNA damage checkpoints. Nature.

[25] Adda di Fagagna F (2008). Living on a break: cellular senescence as a DNA-damage response. Nat Rev Cancer.

[26] Rassoolzadeh H, Bohm S, Hedstrom E, Gad H, Helleday T, Henriksson S, Farnebo M (2016). Overexpression of the scaffold WD40 protein WRAP53beta enhances the repair of and cell survival from DNA double-strand breaks. Cell death & disease.

[27] Jascur T, Brickner H, Salles-Passador I, Barbier V, El Khissiin A, Smith B, Fotedar R, Fotedar A (2005). Regulation of p21(WAF1/CIP1) stability by WISp39, a Hsp90 binding TPR protein. Mol Cell.

[28] Chen X, Chi Y, Bloecher A, Aebersold R, Clurman BE, Roberts JM (2004). N-acetylation and ubiquitin-independent proteasomal degradation of p21(Cip1). Mol Cell.

[29] Sheaff RJ, Singer JD, Swanger J, Smitherman M, Roberts JM, Clurman BE (2000). Proteasomal turnover of p21Cip1 does not require p21Cip1 ubiquitination. Mol Cell.

[30] Jain BP, Pandey S (2018). WD40 repeat proteins: signalling scaffold with diverse functions. Protein J.

[31] Sieben CJ, Sturmlechner I, van de Sluis B, van Deursen JM (2018). Two-Step Senescence-Focused Cancer Therapies. Trends Cell Biol.

