# Supplementary figures and images for: Suppression of TCAB1 expression induced cellular senescence by lessening proteasomal degradation of p21 in cancer cells
Source: Cancer Cell Int. 2021 Jan 7;21:26. doi: 10.1186/s12935-020-01745-3 (PMC7788802; doi:10.1186/s12935-020-01745-3)

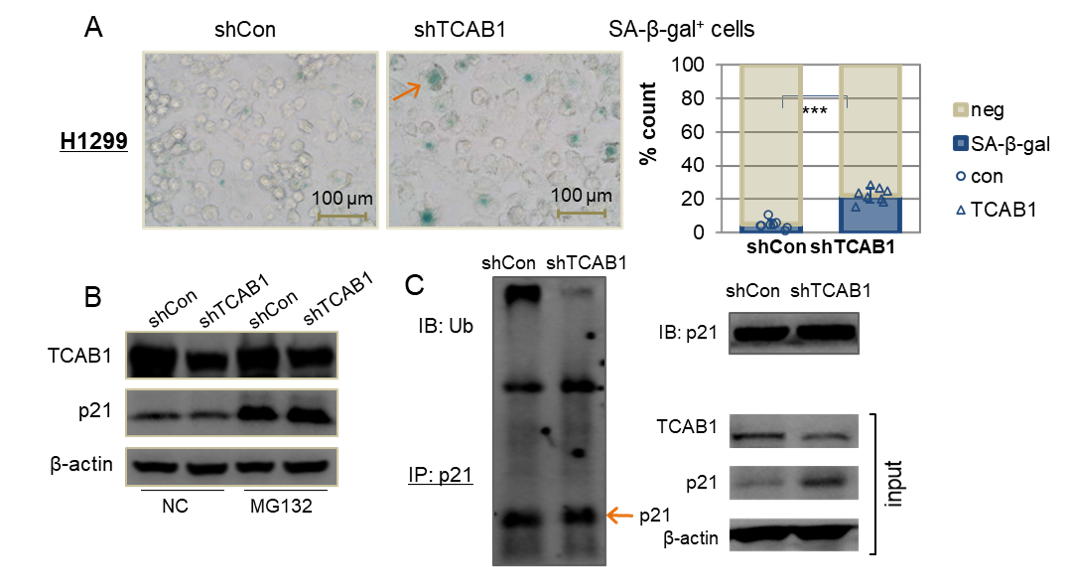

Supplement: Supplementary file 1 — Additional file 1. Fig.S1 A-C [file 12935_2020_1745_MOESM1_ESM.tif]
